# Supplementary figures and images for: An Indo-Pacific damselfish (Neopomacentrus cyanomos) in the Gulf of Mexico: origin and mode of introduction
Source: PeerJ. 2018 Feb 7;6:e4328. doi: 10.7717/peerj.4328 (PMC5807916; doi:10.7717/peerj.4328)

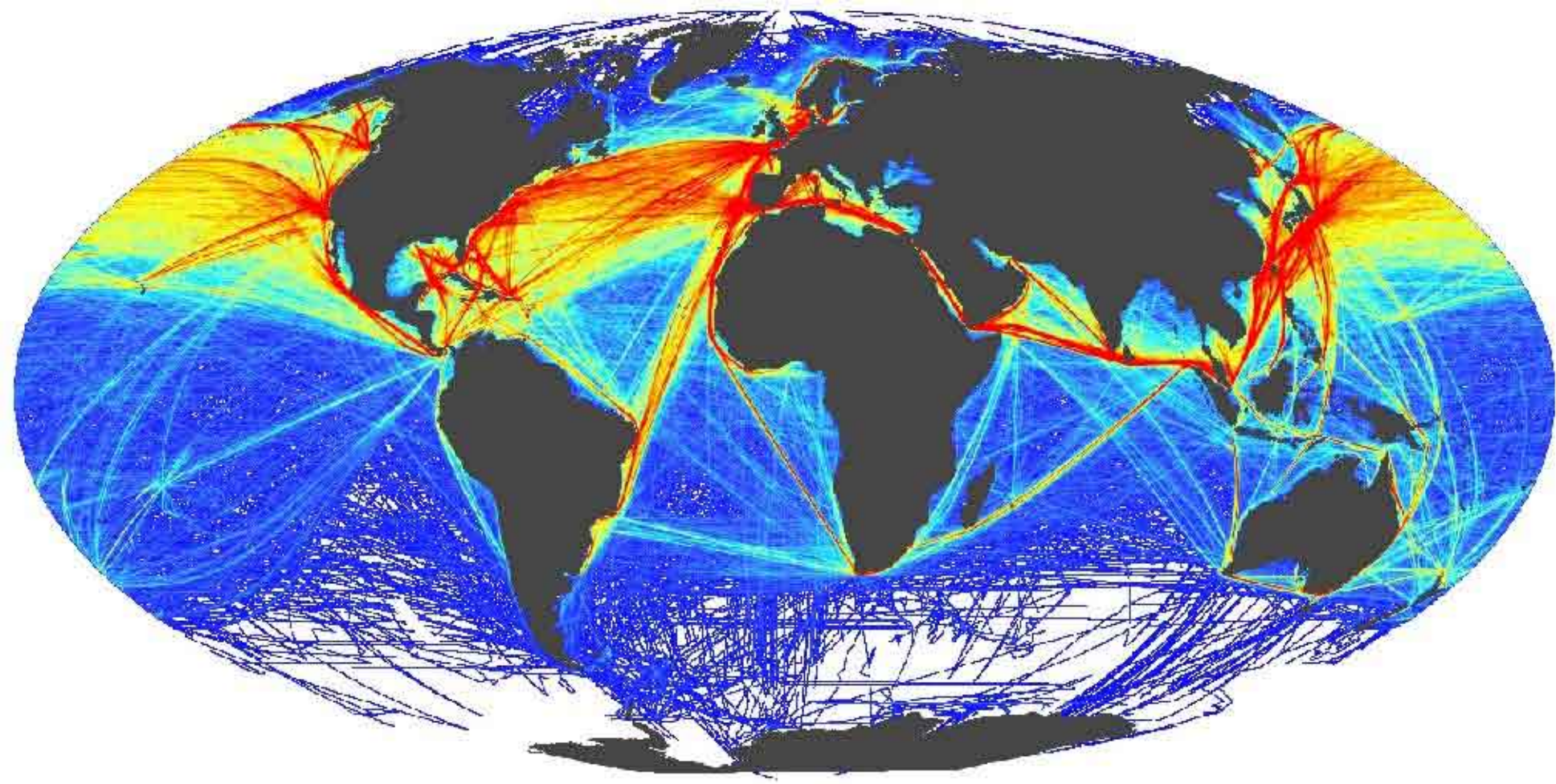

Supplement: Supplemental Information 1 — Global Shipping Routes (from Halpern et al. 2008. A Global Map of Human Impact on Marine Ecosystems.Science 319: 948, DOI: 10.1126/science.1149345, Figure S2, with permission from AAAS). Red routes are the most heavily travelled. [file peerj-06-4328-s001.pdf]
