# Supplementary material for: An Indo-Pacific damselfish (Neopomacentrus cyanomos) in the Gulf of Mexico: origin and mode of introduction
Source: PeerJ. 2018 Feb 7;6:e4328. doi: 10.7717/peerj.4328 (PMC5807916; doi:10.7717/peerj.4328)
Supplement: Table S1 — Sample sites, sample sizes and data for DNA sequences of Neopomacentrus cyanomos and N. taeniurus referred to in this paper. [file peerj-06-4328-s002.docx]

| Location | Sample Site | Sample Site Latitude/Longitude | No. Sequences | Genbank  Accession Nos. | BIN | Voucher collection |
| --- | --- | --- | --- | --- | --- | --- |
| Gulf of Mexico* | Cayo Arcas | 20.21/-91.98 | 63 | MG20477-MG204839 | ADA4740 | N/A |
|  | Madagascar Reef | 21.44/-90.28 | 2 | KUO52527-8 | ADA4740 | CIRR-YUC-PEC-348-A-B |
| Philippines*  (*N. taeniurus*) | Aquarium trade | N/A | 9 | MG220251, MG220258, MG220273, MG220289, MG220300, MG220309-10  MG220317, MG220323 | ACZ4824 | N/A |
| Taiwan | Keelung | 25.150914/121.777 | 2 | KU944331, KX421780 | ADA4740 | ASIZP0073126, ASIZP0912869 |
| Indonesia* | Flores Is., Komodo, | -8.5113/119.724 | 2 | MG220262, MG220291 | ADA4740 | N/A |
| Indonesia* | Irian Jaya, FakFak | -2.8309/131.979 | 3 | MG220249, MG220261  MG220269 | ABX6100 | N/A |
| Papua New Guinea* | Alotau | 10.5266/150.6774 | 2 | MG220257, MG220293 | ABX6100 | N/A |
| Solomon Islands | Guadalcanal, Aruliho | -9.275/159.745 | 4 | MG220259, MG220267  MG220276, MG220287 | ABX6100 | N/A |
| E. Australia | Lizard Is. | -14.6753/145.442 | 1 | MG407348 | ABX6100 | AMS I.41397-008 |
|  | Lizard Is. | -14.686/145.441 | 2 | KP193991, KP194522 | ABX6100 | WAM P.33525.003 |
|  | Coral Sea | -16.716/146.07 | 5 | MG220318, MG220255  MG220274, MG220280  MG220322 | ABX6100 | N/A |
| W. Australia | Montebello Is. | -20.44/115.53 | 4 | MG220272, MG220275  MG220306, MG220324 | ABX6100 | N/A |
| Sri Lanka | N/A | N/A | 5 | MG220254, MG220270 MG220284, MG220301 MG220320 | ACG5999 | N/A |
| India | Gujarat, Veraval | 20.893/70.388 | 2 | MG220297, MG220271 | ACG5999 | CIFE-VRL-NC1 & 2 |
|  | Gujarat, Veraval | 20.893/70.389 | 1 | MG220278 | ADA4740 | CIFE-VRL-NC3 |
| Oman* | Muscat, Fahal Is. | 23.679/58.503 | 6 | MG220256, MG220260 MG220283, MG220299 MG220305, MG220319 | ACG5999 | N/A |
| Saudi Arabia | North of Shuma | 16.753/41.605 | 1 | MG220308 | AAD0615 | CAS236343 |
| Djibouti | Goubbet Kharab | 11.52/46.61 | 4 | MG220250, MG220277  MG220281, MG220307 | AAD0615 | CAS235053, CAS235058 |
| Somaliland | N/A | N/A | 2 | MG220268, MG220304 | AAD0615 | CAS239116, CAS239117 |
| Seychelles | Mahe | -4.6858/55.4355 | 2 | MG700315,MG700316 | AAD0615 | SAIAB76454, SAIAB78251 |
| Madagascar | Nosy Be | -13.7072/48.608 | 5 | JF435057-9, JQ350144-5 | ABX6100 | NBE0240-NBE0244 |
| Mozambique | South | -22.506/35.335 | 1 | MF120962 | ABX6100 | N/A |

**Notes.** Two GenBank records purported to be of *N. cyanomos* are erroneous: KX346261 is a contamination, and JQ729310 represents *N. sororius* Randall & Allen, 2005. Populations for which we obtained DNA sequences from tissue samples we collected or where provided by third parties are indicated with a *; all other sequences were obtained from FISH-BOL.
