# Supplementary material for: An Indo-Pacific damselfish (Neopomacentrus cyanomos) in the Gulf of Mexico: origin and mode of introduction
Source: PeerJ. 2018 Feb 7;6:e4328. doi: 10.7717/peerj.4328 (PMC5807916; doi:10.7717/peerj.4328)
Supplement: Appendix I — Sequences of 3 confirmed Neopomacentrus taeniurus (Bleeker, 1856) [file peerj-06-4328-s003.docx]

Papua New Guinea (ga16nt370)
CCTTTATCTAGTATTTGGTGCCTGAGCTGGAATAGTAGGCACAGCCTTAAGCCTTCTAATTCGAGCAGAACTAAGCCAACCAGGTGCACTATTAGGGGATGACCAGATCTATAATGTTATTGTAACCGCACATGCTTTCGTAATAATTTTCTTTATAGTAATGCCAATTCTGATCGGTGGGTTCGGGAACTGACTGGTACCCCTAATGCTTGGTGCCCCAGACATGGCATTCCCCCGAATAAACAACATAAGCTTCTGGCTCCTCCCCCCGTCATTCCTTCTTCTTCTAGCTTCCTCTGGAGTAGAGGCAGGGGCTGGGACAGGCTGAACTGTCTACCCCCCACTATCCGGCAATCTAGCCCACGCGGGAGCTTCAGTGGACCTAACCATTTTTTCTCTCCACTTAGCAGGGATTTCATCAATCCTCGGAGCAATCAACTTTATCACCACCATTATTAACATAAAACCCCCCGCCATGACCCAGTATCAAACCCCCCTATTCGTCTGGGCCGTCCTAATCACTGCTGTCCTCCTCCTCCTGTCCCTTCCAGTCTTAGCTGCAGGAATTACCATGCTTCTAACAGACCGAAACCTAAACACCACATTCTTCGACCCTGCGGGCGGGGGGGACCCAATCCTTTACCAACACCTT

Papua New Guinea (ga16nt260) CCTTTATCTAGTATTTGGTGGCTGAGCTGGAATAGTAGGCACAGCCTTAAGCCTTCTAATTCGAGCAGAACTAAGCCAACCAGGTGCACTATTAGGGGATGACCAGATCTATAATGTCATTGTAACCGCACATGCTTTCGTAATAATTTTCTTTATAGTAATACCAATTTTGATCGGCGGGTTCGGGAATTGACTAGTACCCCTAATGCTTGGTGCCCCAGACATGGCATTCCCCCGAATAAACAACATAAGCTTCTGGCTCCTCCCTCCGTCATTCCTCCTTCTTCTAGCTTCCTCTGGCGTAGAGGCAGGGGCTGGGACAGGCTGAACTGTCTACCCCCCACTATCCGGCAATCTAGCCCACGCTGGAGCTTCAGTGGACCTAACCATTTTTTCTCTCCACTTGGCAGGAATTTCATCAATCCTCGGGGCAATCAACTTTATTACCACCATTATTAACATAAAACCCCCCGCCATGACCCAGTATCAAACCCCCCTATTCGTCTGGGCTGTCCTAATCACTGCTGTCCTCCTTCTCCTGTCCCTTCCAGTCTTAGCTGCAGGAATTACCATGCTTCTAACAGACCGAAACCTAAACACCACATTCTTCGACCCTGCGGGCGGGGGGGACCCAATCCTTTACCAACACCTT

Indonesia, Raja Ampat (mve1674nb)
CCTTTATCTAGTATTTGGTGCCTGAGCTGGAATAGTAGGCACAGCCTTAAGCCTTCTAATTCGAGCAGAACTAAGCCAACCAGGTGCACTATTAGGGGATGACCAGATCTATAATGTCATTGTAACCGCACATGCTTTCGTTATAATTTTCTTTATAGTAATACCAATTTTGATCGGCGGGTTCGGGAATTGACTAGTACCCCTAATGCTTGGTGCCCCAGACATGGCATTCCCCCGAATAAACAACATAAGCTTCTGGCTCCTCCCTCCGTCATTCCTCCTTCTTCTAGCTTCCTCTGGCGTAGAGGCAGGGGCTGGGACAGGCTGAACTGTTTACCCCCCACTATCCGGCAATCTAGCCCACGCTGGAGCTTCAGTGGACCTAACCATTTTTTCTCTCCACTTAGCAGGGATTTCATCAATCCTCGGGGCAATCAACTTTATTACCACCATTATTAACATAAAACCCCCCGCCATGACCCAGTATCAAACCCCCCTATTCGTCTGGGCTGTCCTAATCACTGCTGTCCTCCTTCTCCTGTCCCTTCCAGTCTTAGCTGCAGGAATTACCATGCTTCTAACAGACCGAAACCTAAACACCACATTCTTCGACCCTGCGGGCGGGGGGGACCCAATCCTTTACCAACACCTT
